# Perceived neighborhood environmental attributes associated with adults’ transport-related walking and cycling: Findings from the USA, Australia and Belgium

**DOI:** 10.1186/1479-5868-9-70

**Published:** 2012-06-12

**Authors:** Delfien Van Dyck, Ester Cerin, Terry L Conway, Ilse De Bourdeaudhuij, Neville Owen, Jacqueline Kerr, Greet Cardon, Lawrence D Frank, Brian E Saelens, James F Sallis

**Affiliations:** 1 Department of Movement and Sport Sciences, Ghent University, Watersportlaan 2, 9000, Ghent, Belgium; 2 Research Foundation Flanders (FWO), Brussels, Belgium; 3 Institute of Human Performance, The University of Hong Kong, Pokfulam, Hong Kong; 4 Graduate School of Public HealthSan, Diego State University, San Diego, CA, USA; 5 Baker IDI Heart and Diabetes Institute, Melbourne, The University of Queensland, Brisbane, Australia; 6 Department of Family and Preventive Medicine, University of California San Diego, La Jolla, CA, USA; 7 School of Community and Regional Planning, University of British Columbia, Vancouver, BC, Canada; 8 Seattle’s Children Hospital Research Institute, University of Washington, Seattle, WA, USA; 9 Department of Psychology, San Diego State University, San Diego, CA, USA

**Keywords:** Physical activity, Ecological model, NEWS, Walkability

## Abstract

**Background:**

Active transportation has the potential to contribute considerably to overall physical activity levels in adults and is likely to be influenced by neighborhood-related built environment characteristics. Previous studies that examined the associations between built environment attributes and active transportation, focused mainly on transport-related walking and were conducted within single countries, limiting environmental variability. We investigated the direction and shape of relationships of perceived neighborhood attributes with transport-related cycling and walking in three countries; and examined whether these associations differed by country and gender.

**Methods:**

Data from the USA (Baltimore and Seattle), Australia (Adelaide) and Belgium (Ghent) were pooled. In total, 6,014 adults (20–65 years, 55.7% women) were recruited in high-/low-walkable and high-/low-income neighborhoods. All participants completed the Neighborhood Environmental Walkability Scale and the International Physical Activity Questionnaire. Generalized additive mixed models were used to estimate the strength and shape of the associations.

**Results:**

Proximity to destinations, good walking and cycling facilities, perceiving difficulties in parking near local shopping areas, and perceived aesthetics were included in a ‘cyclability’ index. This index was linearly positively related to transport-related cycling and no gender- or country-differences were observed. The ‘walkability’ index consisted of perceived residential density, land use mix access, proximity of destinations and aesthetics. A non-linear positive relationship with transport-related walking was found. This association was stronger in women than in men, and country-specific associations were identified: the strongest association was observed in Seattle, the weakest in Adelaide. In Ghent, the association weakened at higher levels of walkability.

**Conclusions:**

For cycling, consistent correlates were found in the three countries, but associations were less straightforward for transport-related walking. Moreover, the identified neighborhood environmental correlates were different for walking compared to cycling. In order to further clarify the shape of these associations and reach more specific international guidelines for developing walkable and bikeable neighborhoods, future studies should include even more countries to maximize environmental variability.

## Background

Physical activity (PA) reduces risk of major chronic diseases, including type 2 diabetes, coronary heart disease and breast and colon cancer; however, a large proportion of the adult population in developed countries does not engage in sufficient PA for health benefits [1]. Improved interventions are required. Ecological models of health behavior emphasize intervening at multiple levels, from individual and social levels of influence, to environmental and policy levels [2]. Research is particularly needed to guide the development of environmental and policy initiatives that increase the convenience and accessibility of PA opportunities, which are expected to have the widest reach and most sustained effects.

Active transportation via walking and cycling has the potential to contribute considerably to overall PA levels of adults and is likely to be modifiable by neighborhood built environment changes. Many trips start or end at home and thus have a significant component within the home neighborhood. Consistent positive associations have been documented between objectively-assessed neighborhood walkability attributes (including residential density, street connectivity, and mixed land use) and transport-related walking and cycling in adults [3-8]. As well as objectively-assessed environmental attributes, residents’ perceptions of the neighborhood environment have been associated with active transportation. Perceived walkability attributes show consistent positive relations, while findings are less consistent for perceived aesthetics, traffic safety, and the availability and quality of walking and cycling facilities, e.g. sidewalks and bike lanes [4,9-14]. Since most previous studies have been conducted in the USA and Australia, where transport-related walking is low but still more prevalent than transport-related cycling, the current evidence base is much stronger for walking. Consequently, additional research focusing on environmental correlates of cycling is needed. Some studies have also suggested that men and women might be differentially affected by the built environment [15,16], so these issues warrant further investigation.

Almost all studies of relationships between PA and the built environment have been conducted within single countries. Because the within-country variability in built environments and in active transportation is likely to be limited, these studies might underestimate the strength of the associations. Worldwide variation in active transportation is large, with one study of walking and cycling showing a range from about 5% of trips in the USA to 50% in Denmark [17]. One study examined the perceived environmental correlates of overall PA in 11 countries, including common methods and a wide variety in environments [18]. Pooled analyses revealed linear associations between perceived environmental attributes and meeting PA guidelines. The associations were stronger compared to what had been reported in single-country studies, probably because of the inclusion of broader environmental variation. To inform policy and planning for walkable and bikeable communities, more multiple-country studies are needed. Combining data from environmentally and culturally different contexts can help to better understand the generalizability of the direction, strength and shape (dose–response) of relationships between the built environment and PA. Moreover, it is important to focus on specific behaviors, as built environment attributes are associated with specific PA outcomes [6,19,20].

We pooled data from three countries (USA, Australia and Belgium) that used common measures and protocols. Analyses were conducted to examine the direction, strength and shape of the associations of perceived neighborhood attributes with transport-related cycling and walking. In addition, we investigated whether these associations differed by country and study site (city); and, whether they differed by gender.

## Methods

### Procedures and participants

Data from studies conducted in four sites within three countries were pooled: the USA (Neighborhood Quality of Life Study [NQLS] in Seattle-King County and Baltimore-Washington DC regions), Australia (Physical Activity in Localities and Community Environments [PLACE] study in Adelaide), and Belgium (Belgian Environmental Physical Activity Study [BEPAS] in Ghent). Study designs and measures were comparable, and detailed information on the procedures and other results of these studies can be found elsewhere [6-8].

Briefly, in each city participants (20–65 year old adults) were recruited in high- and low-walkable and high- and low-income neighborhoods (32 neighborhoods in NQLS and PLACE; 24 in BEPAS). The neighborhoods were chosen to maximize within-metropolitan area variance in income and walkability. In all areas, neighborhoods consisted of clusters of administrative units (block groups in USA, Census Collectors’ Districts in Australia; statistical sectors in Belgium). These administrative units were the smallest geographical units for which area-level information on income and other aggregate demographic attributes was available.

Neighborhood-level walkability was measured objectively with a Geographic Information Systems (GIS) based walkability index, including three (BEPAS) or four (NQLS and PLACE) environmental attributes previously found to be related to higher likelihood of walking [21]: net residential density, intersection density, land use mix, and retail floor area ratio. In BEPAS, retail floor area was not included because GIS data were unavailable for this variable. Detailed information on the calculation of the walkability index is given elsewhere [21]. Neighborhood-level income was determined using census-based median annual household income data [22-24]. The neighborhood selection procedure resulted in an equal number of neighborhoods (eight for NQLS and PLACE; six for BEPAS) among four types, as follows: high-walkable/high-income, high-walkable/low-income, low-walkable/high-income, low-walkable/low-income.

In the USA sites (NQLS study), data collection took place between May 2002 and June 2005. Adults living in the 32 neighborhoods were randomly selected from lists supplied by a marketing company, then contacted by phone and mailed study materials if they agreed to participate. The mailed survey was completed by 2,199 participants out of 8,504 eligible individuals contacted by telephone (response rate = 25.9%; 1,287 participants in Seattle and 912 participants in Baltimore regions). In Adelaide, Australia (PLACE study), data collection took place between July 2003 and June 2004. A simple random sampling procedure was used to select possible participants within the 32 neighborhoods. Invitation letters and surveys were mailed. In total, 2,650 of the 23,128 contacted adults returned a completed survey (response rate = 11.5%). In Ghent, Belgium (BEPAS study), data collection took place between May 2007 and September 2008. In each neighborhood, 250 randomly selected adults received an invitation letter and were visited at home two-to-six days after posting the letter. In total, 1,165 adults participated in BEPAS (response rate = 58.0%). In all studies, data were collected throughout the year to take seasonal variation into account.

All participants completed a written informed consent form. NQLS was approved by Institutional Review Boards at participating USA academic institutions, PLACE was approved by the Behavioral and Social Sciences Ethics Committee of the University of Queensland, and BEPAS was approved by the Ethics Committee of the Ghent University Hospital.

### Measures

#### Environmental perceptions

To measure perceived neighborhood built- and social-environmental factors, the Dutch and English versions of the previously validated Neighborhood Environmental Walkability Scale (NEWS) were used [9,25-28]. Before data analysis, comparability of the NEWS items across the three countries was assessed by two independent raters. Based on these ratings, only the comparable NEWS items (40 out of 68) were included in the present analyses. Neighborhood environment scales included in the analyses were residential density (5 items), land use mix diversity (proximity of destinations and number of destinations within a 20 min walk; 11 items), land use mix access (3 items), street connectivity (2 items), walking and cycling facilities (6 items), aesthetics (3 items), traffic safety (3 items), and crime safety (3 items). The following were used as single items: ‘parking is difficult near local shopping areas’; ‘there are many barriers in my neighborhood which make it difficult to walk from one place to the other’; ‘distance from home to a public transit stop’ and ‘streets in my neighborhoods do not have many cul-de-sacs’. Calculation of the NEWS subscales and selection of the single items were based on methods proposed by Cerin and colleagues [27] after a cross-validation of the confirmatory factor analysis structure of NEWS. All environmental items were rated on a four-point scale (1–4 from *strongly disagree* to *strongly agree*), except for residential density and land use mix access (five-point scales; 1–5). Scoring details and a digital version of NEWS can be found on http://sallis.ucsd.edu/measures.html. Site-specific descriptive statistics of the NEWS scales are shown in Table 1.

**Table 1:** Site-specific descriptive statistics for all outcome variables, socio-demographic covariates and explanatory variables

	**Seattle regions USA**	**Baltimore regions**	**Adelaide Australia**	**Ghent Belgium**
	**(n=1,287)**	**USA (n=912)**	**(n=2650)**	**(n=1,165)**
**Site region characteristics**^**a**^				
Number of inhabitants	1,931,249	5,773,552	1,289,265	248,269
Area (km^2^)	5,506	25,210	1,827	156
Population density (inhabit/km^2^)	351	229	706	1,589
Mean temperature January (°C)	4.5	2.7	23.1	3.1
Mean temperature July (°C)	18.4	27.6	11.4	17.7
Average precipitation/year (mm)	944.6	1065.3	500.0	820.0
**Area/neighborhood characteristics**				
Area level household income[mean (SD)]	USD 56,680 (19,912)	USD 59,930 (21,758)	USD 37,669 (12,826)	USD 55,240 (5,144)
High walkable areas participants (%)	50.6	49.2	48.6	50.0
High SES areas participants (%)	51.3	52.5	52.2	50.5
**Sample socio-demographic characteristics**				
Gender - % women	45.1	52.3	63.7	52.0
Age [mean (SD)]	44.0 (11.0)	46.6 (10.7)	44.5 (12.3)	42.7 (12.6)
Marital status - % with partner	63.1	60.1	60.3	73.0
Education - % tertiary education	63.0	67.2	45.5	60.3
Driver’s license - % with license	95.6	94.4	89.2	90.2
Number of drivable vehicles[mean (SD)]	2.0 (1.2)	1.9 (1.1)	1.6 (1.0)	1.6 (1.1)
Body mass index [mean (SD)]	26.6 (5.5)	27.2 (5.9)	26.2 (5.9)	24.3 (3.9)
**Physical activity variables** [mean (SD)]				
% doing any transport-related walking	68.1	68.8	74.9	52.4
% doing any transport-related cycling	9.0	6.6	11.2	43.4
Min/week of transport-related walking	143.3 (205.9)	154.2 (215.9)	167.7 (216.8)	63.1 (113.6)
Min/week of transport-related cycling	15.5 (71.9)	22.0 (92.5)	22.0 (92.5)	77.5 (141.3)
Min/week of other physical activity	990.7 (817.5)	1016.4 (863.0)	1332.8 (950.1)	661.1 (540.2)
**Perceived physical environmental attributes** [mean (SD)]				
Residential density	140.5 (49.6)	156.4 (58.2)	143.5 (46.1)	201.0 (79.8)
Land use mix diversity – proximity of				
destinations	3.3 (0.8)	3.1 (0.9)	3.4 (0.7)	3.0 (0.9)
Land use mix diversity - # destinations				
within 20min walk	8.2 (2.8)	7.5 (3.1)	9.2 (3.1)	7.2 (3.3)
Land use mix access	3.2 (0.8)	3.0 (0.8)	3.5 (0.7)	3.1 (0.6)
Not many cul-de-sacs	2.8 (1.1)	2.8 (1.2)	2.8 (1.1)	3.0 (0.8)
Parking difficult near local shopping				
area	1.9 (1.0)	1.8 (0.9)	2.0 (1.0)	2.5 (0.9)
Not many barriers in neighborhood	3.2 (1.0)	3.7 (0.6)	3.6 (0.6)	3.3 (0.7)
Street connectivity	3.0 (0.8)	3.0 (0.8)	3.0 (0.7)	2.8 (0.6)
Proximity to transit stop	3.4 (1.3)	3.4 (1.3)	3.8 (1.2)	3.9 (1.7)
Walking and cycling facilities				
and cycling	2.9 (0.7)	3.1 (0.6)	3.0 (0.5)	2.7 (0.5)
Aesthetics	3.2 (0.7)	3.2 (0.6)	3.2 (0.7)	2.5 (0.6)
Traffic safety	3.4 (0.6)	3.4 (0.7)	3.0 (0.8)	3.1 (0.6)
Crime safety	3.4 (0.6)	3.4 (0.7)	3.0 (0.8)	3.1 (0.6)

SD = standard deviation; USD = United Stated Dollar.

*Note:* all perceived environmental attributes were positively scored: higher score = more walkable. 18.36% of missing observations on at least one variable.

^a^ United States census data 2010, www.census.gov; Australian Bureau of Statistics 2010, www.abs.gov.au; Belgian National Institute of Statistics 2010, www.statbel.fgov.b.

#### Physical activity

Self-reported PA was measured with the International Physical Activity Questionnaire (IPAQ; long, past seven days version; questionnaire available on https://sites.google.com/site/theipaq/questionnaires). In a previous 12-country validation study [29], PA assessed by the IPAQ showed good reliability (intra-class correlations range from .46 to .96) and fair-to-moderate criterion validity compared against accelerometers (median ρ=.30). Frequency (number of days in the last seven days) and duration (minutes/day) of PA in different domains were queried. Based on this information, separate estimates of mins/week of transport-related walking and cycling were calculated by multiplying frequency per week with duration per day. Moreover, so that other forms of PA could be controlled for, mins/week (with no weighting for intensity) were calculated for work-related, household-related and leisure-time PA. In Belgium, the interviewer-administered version of IPAQ was used, while in Australia and the USA, participants completed the self-administered version.

#### Socio-demographic information

Self-reported socio-demographic variables included gender, age, marital status (partner vs. no partner), educational level (college/university degree vs. no college/university degree), body mass index (calculated from height and weight), having a driver’s license (yes/no), and number of drivable vehicles in the household.

### Data analytic plan

Descriptive statistics (means, standard deviations, percentages, and percentage of missing values) were computed by study site for all variables. Generalized additive mixed models (GAMMs) [30] with negative binomial variance and logarithmic link functions estimated the strength and shape of the associations of perceived environmental attributes with weekly minutes of transport-related cycling and walking. GAMMs allow modeling of data with positively skewed distributions (typical of PA data), while accounting for clustering effects arising from a multi-stage sampling strategy. They can also estimate complex, dose–response relationships of unknown form. The shape of dose-repose relationships was estimated using thin plate splines, a method appropriate for the estimation of relationships of unknown, complex shape [30]. Random intercepts were specified to account for dependency in the data arising from the respondents being sampled from selected neighborhoods. The appropriateness of the GAMMs and their link functions was assessed via normal quantile-quantile plots of residuals; plots of model residuals against the model fitted values; and a comparison of the Akaike Information Criteria (AIC) values, whereby a lower AIC is indicative of a better-fitting model [30].

A first set of models estimated the dose–response relationships of single perceived environmental attributes with the two outcomes (transport-related walking and cycling), adjusting for socio-demographic covariates and study site. Separate models were run to estimate main effects of environmental attributes, two-way gender by environmental attributes and study site by environmental attributes interaction effects, and three-way gender by environmental attributes by study site interaction effects. All perceived environmental attributes with main and/or interaction effects significant at a 0.15 probability level were included in a multiple-predictor model of transport-related cycling or transport-related walking. The average absolute value of the bivariate correlation coefficients among perceived environmental attributes was 0.16, with the highest correlation observed between land use mix – diversity (proximity to destinations) and land use mix – access (*r* = 0.54). Exclusion of one of the latter attributes from the multiple predictor models yielded changes in the respective regression coefficients smaller than 10%. Hence, multicollinearity was not a problem in this study. The main and interaction terms that remained significant at a 0.15 probability level were retained in a final model [31]. We adopted a 0.15 probability level because the findings from this study were based only on data from three Western countries and, thus, from an international perspective, are still somewhat exploratory. Namely, it is possible that some of the environmental features that were only weakly correlated with the outcomes might show stronger relationships in other geographical locations or other samples. These significant variables were also used to construct a composite environmental index for each outcome variable representing the sum of the standardized scores (z-scores, calculated for the total sample) of the variables that were linearly positively related with the outcome. For variables that showed a curvilinear relationship, z-scores were computed using appropriate polynomial functions best describing the relationship (i.e. the sum of the linear and quadratic values of a z-score weighted by their respective regression coefficients constrained to sum to 1, and derived from a GAMMs including a linear and a quadratic term for a predictor of interest). Two composite indices were created – namely, a cyclability and a walkability index. The dose–response relationships of these two indices with the relative outcomes were estimated using GAMMs (see above).

All models were adjusted for area-level household income (deciled at the country-level) to minimize bias in statistical estimators due to the adoption of an unequal probability sampling design for the selection of neighborhoods [32]. Specifically, area-level household income and objectively-measured walkability determined the probability of selecting specific neighborhoods. Objectively-measured walkability was not included in the models because it was substantially related to the perceived environmental attributes being examined. Thus, the inclusion of these perceived attributes in the regression models addressed the possible bias induced by the unequal selection probabilities based on neighborhood walkability.

There were approximately 18% of cases with missing values on at least one of the variables. The likelihood of having missing data was positively related to age (p < .001) and weekly minutes of walking for transport (p < .01), but negatively related to area-level household income, educational attainment, perceived safety from traffic, and perceived safety from crime (p < .001). Given that data were missing at random (MAR; i.e., the probability that a variable is missing was related to other observed data) rather than missing completely at random (MCAR), 10 multiple imputed datasets were created, as recommended by Rubin [33]. Conducting complete-case analyses when data are MAR would yield biased results [33]. Imputations were performed using chained equations whereby separate models were constructed for each variable with missing values (depending on their level of measurement and distributional assumptions). The variables entered in each model were those involved in the planned analyses. All analyses were conducted in R [34] using the packages ‘car’ [35], ‘mgcv’ [30] and ‘Design’ [36].

## Results

Table 1 reports the descriptive statistics for each study site including region characteristics, sample socio-demographic characteristics, perceived environmental attributes, and transport-related PA. The total sample consisted of 6,014 participants; 55.7% were women, 63.3% were living with a partner, 55.4% had tertiary education and 91.6% had a driver’s license. Mean age of the total sample was 44.4 yrs (SD = 11.9); mean body mass index (BMI) was 26.1 kg/m^2^ (SD = 5.5).

### Socio-demographic correlates of transport-related cycling and walking

Table 2 reports the associations of socio-demographic covariates with weekly minutes of transport-related cycling and walking, unadjusted for perceived environmental attributes. Belgian participants reported significantly more minutes of cycling and fewer minutes of walking than did participants from the other study sites. Australian respondents reported higher levels of cycling than did their American counterparts. Being a woman, living in higher income areas, having a driver’s license and higher BMI were predictive of lower levels of transport-related cycling. Having a partner and a driver’s license were predictive of lower levels of transport-related walking. Age showed a curvilinear relationship with both outcome variables (figures not shown). An inverted-U relationship was observed between age and walking for transport, whereby younger and older respondents showed equally higher levels of walking than did those aged 30–50 years (Table 2). For the relationship between age and transport-related cycling, similar levels of transport-related cycling were found in respondents up to 40 years of age, but a negative association was identified for those aged 40+ years.

**Table 2:** Associations of socio-demographic covariates with transport-related cycling and walking

**Variables**	**Cycling (min/wk)**			**Walking (min/wk)**		
	**exp(*b*)**	**exp (95% CI)**	***p***	**exp(*b*)**	**exp (95% CI)**	***p***
Area-level household income (deciles)	0.959	0.922, 0.997	.033	0.980	0.957, 1.004	.106
Gender (Men vs. Women)	0.442	0.360, 0.543	<.001	0.963	0.892, 1.040	.339
Age (yrs)						
Linear component	1.062	0.999, 1.130	.055	0.949	0.927, 0.971	<.001
Quadratic component	0.999	0.998, 1.000	.016	1.001	1.000, 1.001	<.001
Marital status (without vs. with partner)	0.873	0.704, 1.083	.218	0.899	0.829, 0.975	.010
Tertiary education (no vs. yes)	1.056	0.852, 1.308	.619	0.972	0.895, 1.057	.511
Holder of a driver’s license						
(no vs. yes)	0.511	0.347, 0.753	<.001	0.579	.500, 0.671	<.001
Body mass index (kg/m^2^)	0.953	0.935, 0.972	<.001	0.994	0.987, 1.002	.124
Study site (reference category: Ghent, Belgium)						
Seattle, USA	0.203	0.147, 0.279	<.001	2.325	1.772, 3.051	<.001
Baltimore, USA	0.143	0.100, 0.204	<.001	2.604	1.979, 3.424	<.001
Adelaide, Australia	0.363	0.275, 0.478	<.001	2.703	2.150, 3.398	<.001

*Note.* Associations are adjusted for all other socio-demographic covariates. All regression models used a negative binomial variance function and a logarithmic link function. Exp(*b*) antilogarithm of regression coefficient; exp(95% CI) = antilogarithms of the 95% confidence intervals of the regression coefficient; *p* = probability value. The antilogarithms of the regression coefficients represent the proportional increase (if exp(b) > 1.00) or decrease (if exp(b)<1.00) in the outcome variables associated with a unit increase in an explanatory variable.

### Dose–response associations between perceived physical environmental attributes and transport-related cycling

Main effects models of transport-related cycling with single environmental attributes as predictors (adjusted for socio-demographic confounders) gave support for positive associations with most environmental attributes examined, except for lack of barriers in the neighborhood, aesthetics, traffic safety, and crime safety (Table 3). Gender was a significant moderator of the relationships of transport-related cycling with aesthetics and crime safety, with women showing no significant associations and men showing positive associations. The associations of aesthetics and crime safety with cycling were also moderated by study site, with significant positive associations observed only in Baltimore. Greater parking difficulty near shopping areas was positively associated with transport-related cycling only in the two USA study sites.

**Table 3:** Associations of perceived environmental attributes with transport-related cycling (min/wk)

**Variables**	**exp(*b*)**	**exp (95% CI)**	***p***
**STEP 1: Separate models with single environmental attributes**			
*Main effects*			
Residential density	1.002	1.001, 1.004	.008
Land use mix-diversity – proximity of destinations	1.186	1.071, 1.312	.001
Land use mix-diversity – # destinations within 20min walk	1.182	1.067, 1.310	.001
Land use mix-access	1.132	1.021, 1.256	.019
Not many cul-de-sacs	1.110	1.010, 1.220	.032
Parking difficult near local shopping areas	1.145	1.036, 1.266	.008
Not many barriers in neighborhood	1.101	0.972, 1.248	.130
Street connectivity	1.175	1.063, 1.300	.002
Proximity of transit stop	1.088	1.001, 1.183	.047
Walking and cycling facilities	1.152	1.040, 1.275	.007
Aesthetics	1.045	0.932, 1.172	.454
Traffic safety	0.981	0.843, 1.142	.130
Crime safety	1.042	0.893, 1.217	.603
*Interaction effects*			
Gender by Aesthetics			
Association in men	1.471	1.170, 1.850	<.001
Association in women	0.874	0.714, 1.069	.190
Gender by Crime safety			
Association in men	1.350	1.078, 1.690	.009
Association in women	0.901	0.745, 1.089	.280
Site by Parking difficult near local shopping areas			
Association in Ghent, Belgium	1.058	0.842, 1.329	.627
Association in Seattle, USA	1.267	1.016, 1.580	.036
Association in Baltimore, USA	1.564	1.198, 2.042	<.001
Association in Adelaide, Australia	1.038	0.900, 1.196	.609
Site by Aesthetics			
Association in Ghent, Belgium	1.120	0.786, 1.598	.531
Association in Seattle, USA	1.153	0.835, 1.592	.386
Association in Baltimore, USA	2.251	1.454, 3.482	<.001
Association in Adelaide, Australia	0.875	0.694, 1.102	.256
Site by Crime safety			
Association in Ghent, Belgium	1.040	0.709, 1.524	.842
Association in Seattle, USA	1.076	0.766, 1.513	.672
Association in Baltimore, USA	1.842	1.230, 2.759	.003
Association in Adelaide, Australia	0.937	0.767, 1.144	.522
STEP 2: Model with multiple environmental attributes and interaction effects *			
Land use mix-diversity – proximity of destinations	1.156	1.013, 1.318	.031
Parking difficult near local shopping areas	1.111	1.001, 1.232	.046
Walking and cycling facilities	1.295	1.061, 1.582	.011
Gender by Aesthetics			
Association in men	1.593	1.245, 2.038	<.001
Association in women	0.933	0.741, 1.173	.551
Site by Aesthetics			
Association in Ghent, Belgium	1.280	0.894, 1.831	.177
Association in Seattle, USA	0.960	0.690, 1.335	.807
Association in Baltimore, USA	2.195	1.401, 3.439	<.001
Association in Adelaide, Australia	0.818	0.640, 1.045	.108
STEP 3: Final model with composite environmental index of cyclability**			
Index (Land use mix-diversity, proximity of destinations + Parking difficult in near shopping areas + Walking and cycling facilities + Aesthetics)	1.111	1.056, 1.169	<.001

*Note.* Gender, age, living arrangements (with vs. without partner), driver’s license holder (yes vs. no), tertiary education (yes vs. no), area household income (in deciles), body mass index, study site, and weekly minutes of other types of physical activity (household, work and leisure) were included as covariates in all models. All regression models used a negative binomial variance function and a logarithmic link function. Only significant interaction effects are presented. Exp(*b*) antilogarithm of regression coefficient; exp(95% CI) = antilogarithms of the 95% confidence intervals of the regression coefficient; *p* = probability value; * = final model including only predictors significant at p<.15; **= final model including cyclability index based on environmental attributes independently positively related to cycling. The antilogarithms of the regression coefficients represent the proportional increase (if exp(b) > 1.00) or decrease (if exp(b)<1.00) in average min/wk of transport-related cycling associated with a unit increase in a perceived environmental attribute.

The multiple-predictor model of transport-related cycling yielded significant positive independent associations for proximity of destinations, walking and cycling facilities, and difficulties in parking near local shopping areas. The moderating effects of gender and study site by aesthetics remained significant.

A "cyclability index" was constructed based on the above findings. It consisted of the sum of the standardized scores (z-scores) of perceived environmental attributes independently positively related (overall, within a site or socio-demographic subgroup) to transport-related cycling. These were proximity to destinations, walking and cycling facilities, difficulties in parking near local shopping areas, and aesthetics. The index was linearly positively related to transport-related cycling, with one unit difference in the index being predictive of an 11.1% difference in transport-related cycling (last model in Table 3). No significant interaction effects of socio-demographic factors with the cyclability index were observed.

### Dose–response associations between perceived physical environmental attributes and transport-related walking

**Figure 1:**
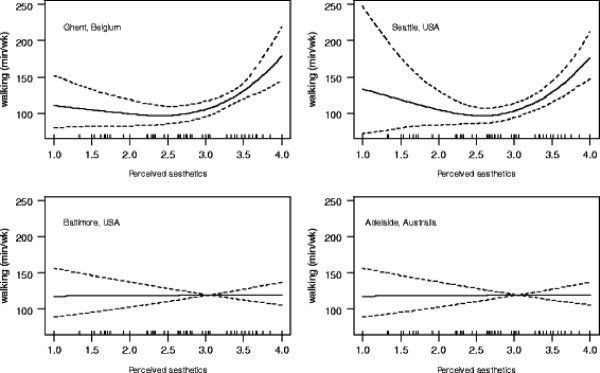
Dose–response relationship of perceived neighborhood aesthetics with weekly minutes of transport-related walking by study site.

Main effect models with single environmental attributes as predictors indicated positive relationships between transport-related walking and most environmental attributes (adjusted for socio-demographic confounders) except for difficulties in parking near shopping areas, street connectivity, traffic safety, and crime safety (Table 4). Aesthetics showed a curvilinear relationship; only higher levels of aesthetics (i.e., values from 3 to 4 on a 4-point scale) were positively associated with walking (graphs not shown). Study site moderated this curvilinear relationship, which was significant only for participants from Ghent and Seattle (Figure 1). Study site also moderated the relationships with access to destinations (land use mix-access), average proximity of destinations, and number of destinations within a 20 min walk. The statistical significance of the effect for access to destinations was positive in all study sites, but significantly stronger for the Belgian than the other sites. Both measures of land use mix-diversity (proximity and number of destinations) were significantly related to walking in Ghent and Seattle, and marginally related to walking in Baltimore. Finally, the association of access to destinations with walking was stronger in men than in women.

**Table 4:** Associations of perceived environmental attributes with transport-related walking (min/wk)

**Variables**	**exp(*b*)**	**exp (95% CI)**	***p***
**STEP 1: Separate models with single environmental attributes**			
*Main effects*			
Residential density	1.186	1.072, 1.312	<.001
Land use mix-diversity – proximity of destinations	1.110	1.060, 1.161	<.001
Land use mix-diversity – # destinations within 20min walk	1.076	1.029, 1.126	.001
Land use mix-access	1.229	1.157, 1.305	<.001
Not many cul-de-sacs	1.110	1.010, 1.220	.032
Parking difficult near local shopping areas	1.024	0.984, 1.071	.225
Not many barriers in neighborhood	1.064	1.010, 1.121	.020
Street connectivity	1.035	0.971, 1.105	.290
Proximity of transit stop	1.091	1.051, 1.132	<.001
Walking and cycling facilities	1.047	1.002, 1.092	.037
Aesthetics (linear component)*	1.103	0.994, 1.224	.250
Aesthetics (curvilinear smooth)*	F(4.37)=3.60		.005
Traffic safety	0.973	0.932, 1.015	.200
Crime safety	0.987	0.943, 1.033	.568
*Interaction effects*			
Gender by Land use mix-access			
Association in men	1.299	1.198, 1.408	<.001
Association in women	1.170	1.087, 1.259	<.001
Site by Land use mix-diversity – proximity of destinations			
Association in Ghent, Belgium	1.436	1.261, 1.635	<.001
Association in Seattle, USA	1.179	1.057, 1.316	.003
Association in Baltimore, USA	1.122	0.996, 1.263	.058
Association in Adelaide, Australia	1.052	0.967, 1.145	.237
Site by Land use mix-diversity - # destinations within 20min walk			
Association in Ghent, Belgium	1.078	1.041, 1.116	<.001
Association in Seattle, USA	1.038	1.006, 1.071	.020
Association in Baltimore, USA	1.029	0.996, 1.064	.082
Association in Adelaide, Australia	1.005	0.985, 1.025	.632
Site by Land use mix-access			
Association in Ghent, Belgium	1.465	1.252, 1.716	<.001
Association in Seattle, USA	1.237	1.105, 1.385	<.001
Association in Baltimore, USA	1.268	1.108, 1.451	<.001
Association in Adelaide, Australia	1.139	1.040, 1.248	.005
Site by Aesthetics (linear component)*			
Association in Ghent, Belgium	1.160	0.867, 1.552	.317
Association in Seattle, USA	1.153	0.827, 1.607	.400
Association in Baltimore, USA	1.005	0.894, 1.129	.937
Association in Adelaide, Australia	1.030	0.959, 1.106	.421
Site by Aesthetics (curvilinear smooth)*			
Association in Ghent, Belgium	F(2.50)=3.64		.018
Association in Seattle, USA	F(2.81)=6.92		<.001
Association in Baltimore, USA	F(0.67)=0.01		.840
Association in Adelaide, Australia	F(1.78)=0.21		.789
**STEP 2: Model with multiple environmental attributes and interaction effects**^**#**^			
Residential density	1.003	1.002, 1.003	<.001
Gender by Land use mix-access			
Association in men	1.248	1.182, 1.318	<.001
Association in women	1.112	1.038, 1.213	.004
Site by Land use mix-diversity – proximity of destinations			
Association in Ghent, Belgium	1.351	1.267, 1.440	<.001
Association in Seattle, USA	1.066	0.995, 1.191	.253
Association in Baltimore, USA	1.028	0.912, 1.159	.652
Association in Adelaide, Australia	0.981	0.900, 1.070	.667
Site by Aesthetics (linear component)*			
Association in Ghent, Belgium	1.081	0.829, 1.410	.565
Association in Seattle, USA	1.094	0.804, 1.488	.568
Association in Baltimore, USA	1.022	0.912, 1.146	.708
Association in Adelaide, Australia	1.014	0.945, 1.087	.706
Site by Aesthetics (curvilinear smooth)*			
Association in Ghent, Belgium	F(2.28)=4.27		.011
Association in Seattle, USA	F(3.23)=4.40		.003
Association in Baltimore, USA	F(0.69)=0.21		.555
Association in Adelaide, Australia	F(1.13)=0.12		.760
**STEP 3: Models with composite environmental index of walkability**^**##**^			
*Main effect*			
Index (Residential density + Land use mix-access+ Land use mix-diversity, proximity of destinations + Aesthetics: linear and quadratic terms)			
Linear component*	1.276	1.218, 1.336	<.001
Curvilinear smooth*	F(1.78)=34.85		<.001
*Interaction effects*			
Gender by Index (linear component)*			
Association in men	1.270	1.033, 1.561	.024
Association in women	1.248	1.181, 1.319	<.001
Gender by Index (curvilinear smooth)*			
Association in men	F(3.09)=17.83		<.001
Association in women	F(0.81)=79.63		<.001
Site by Index (linear component)*			
Association in Ghent, Belgium	1.179	1.136, 1.223	<.001
Association in Seattle, USA	1.124	1.085, 1.163	<.001
Association in Baltimore, USA	1.080	1.037, 1.125	<.001
Association in Adelaide, Australia	1.054	1.026, 1.083	<.001
Site by Index (curvilinear smooth)*			
Association in Ghent, Belgium	F(2.50)=35.32		<.001
Association in Seattle, USA	F(1.00)=45.58		<.001
Association in Baltimore, USA	F(1.00)=14.26		<.001
Association in Adelaide, Australia	F(1.00)=14.09		<.001

*Note.* Gender, age, living arrangements (with vs. without partner), driver’s license holder (yes vs. no), tertiary education (yes vs. no), area household income (in deciles), body mass index, study site, and weekly minutes of other types of physical activity (household, work and leisure) were included as covariates in all models. All regression models used a negative binomial variance function and a logarithmic link function. Only significant interaction effects are presented. Exp(*b*) antilogarithm of regression coefficient; exp(95% CI) = antilogarithms of the 95% confidence intervals of the regression coefficient; *p* = probability value; * for significant curvilinear relationships, the significance of both linear component and curvilinear smooth are reported; ^#^ = final model including only predictors significant at p<.15; ^##^ = final models including walkability index based on environmental attributes independently positively related to walking. The antilogarithms of the regression coefficients represent the proportional increase (if exp(b) > 1.00) or decrease (if exp(b)<1.00) in average min/wk of transport-related walking associated with a unit increase in a perceived environmental attribute.

The final model for transport-related walking with multiple environmental predictors resulted in a significant positive main effect of residential density. The site-by-aesthetics interaction effect remained significant (with continued notable curvilinear effects), as did that for the gender by access to destinations interaction. Although the overall site by proximity of destinations interaction effect was also significant, it resulted in only one (rather than two) study sites showing a significant association (Table 4).

**Figure 2:**
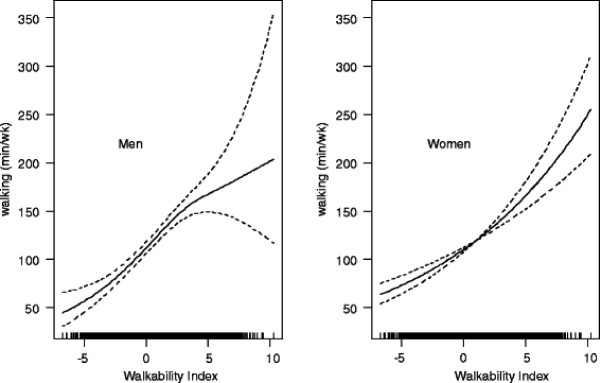
Dose–response relationship of perceived Walkability Index with weekly minutes of transport-related walking by gender.

**Figure 3:**
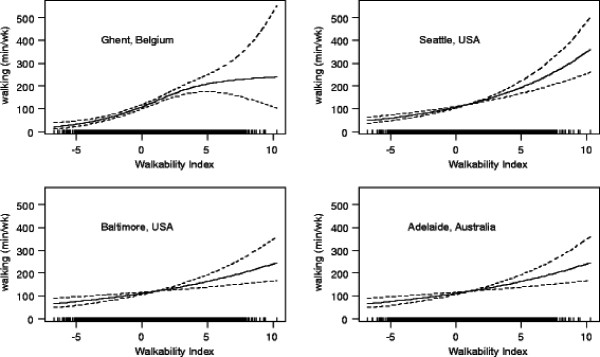
Dose–response relationship of perceived Walkability Index with weekly minutes of transport-related walking by study site.

A composite walkability index of perceived environmental correlates of transport-related walking was computed. It consisted of the sum of the standardized scores (z-scores) of environmental attributes showing an independent linear positive relationship with walking in the whole sample or one of the subsamples (residential density, land use mix-access, proximity of destinations) and the quadratic polynomial of the z-score of aesthetics (describing the shape of the observed relationship between aesthetics and walking in one of the sites). Overall, the index was positively non-linearly related to walking for transport. This relationship was stronger in women than men (Figure 2). For women, the steepness of the dose–response curve was positively associated with the index (i.e., the slope gradually increased with higher index values). In men, the steepness of the curve decreased at above average values of the index (i.e., at a walkability index value of ~3). The strongest relationship between the walkability index and transport-related walking was observed for Seattle, and the weakest for Adelaide (Table 4). For Ghent, the association weakened at the higher levels of walkability (Figure 3).

## Discussion

This study examined dose–response associations of perceived built environment attributes with transport-related walking and cycling in adult samples from metropolitan areas in the USA, Australia and Belgium. After controlling for socio-demographic covariates, the associations with the outcome variables were in the expected direction and for transport-related walking several site- and gender-specific interactions were identified. Moreover, the built environment correlates of transport-related walking were different than the factors related to cycling, supporting the need for a behavior-specific focus [2,6,19,20].

Proximity of destinations, availability and quality of walking and cycling facilities, aesthetics, and perceiving difficulties parking near local shopping areas were included in a composite index of correlates of transport-related cycling (cyclability index). The index showed a positive association with cycling, with an increase of approximately 11% in transport-related cycling per unit increase in the index. The model provided evidence of a linear gradient in the association with transport-related cycling, so the more supportive the environment on these four environmental characteristics, the more time an adult spent cycling for transportation. Present results are partly in line with the limited evidence of previous studies. Bicycling facilities are valued by bicycle commuters [37], and they appear to be especially effective in combination with other interventions, like supportive land use planning and restrictions on car use [38]. Previous studies found land use mix to be positively related to transport-related cycling [11,14,25], but mixed evidence has been found concerning the role of aesthetics [14,39,40]. It has been suggested that aesthetics might relate more-strongly with recreational PA [20]; adults possibly attach more importance to aesthetic-related features for activities they choose to do during their leisure-time. However, the present results indicated that perceiving an environment as aesthetically pleasing can contribute to explaining transport-related cycling as well. Perceiving difficulties in being able to park near local shopping areas has not been examined as a separate item before, but it emerged in the present study as a significant facilitator of cycling.

For the cyclability index, no interactions with gender and study site were found. The environmental perceptions included in the studies were similarly related to cycling in three countries with large variations in cycling prevalence and environmental characteristics. Since cycling rates are much lower in the USA and Australia than in Belgium, efforts to increase cycling rates in those countries might apply similar approaches to what has been done in Ghent (Belgium). In Ghent, the activity-friendliness of the city centre has been increased by prohibiting car traffic and improving bike lanes and sidewalks [41]. Recently, some USA cities (e.g. Portland, Minneapolis) have also implemented policies and programs to encourage more cycling and to make cycling safer. Although cycling rates are still low compared with European cities, strategies like providing more and better bike lanes, installing bike boxes with advance stop lines for cyclists at intersections, offering bike parking and introducing bicycle-sharing programs have led to growing cycling levels in these cities [42].

For transport-related walking, the associations were less straightforward. Residential density, land use mix-access, proximity to destinations and aesthetics were included in the perceived ‘walkability’ index and showed positive associations with transport-related walking, but gender and study-site interactions were identified. The significant associations with perceived walkability characteristics (i.e. residential density and land use mix factors) confirmed previous findings identifying these walkability attributes as consistent correlates of transport-related walking [4,13,14,20,25,43]. The multiple predictor model showed a curvilinear association between perceived aesthetics and transport-related walking. This curvilinear association was only significant in Belgium and Seattle, and showed a steep increase in walking when the score for aesthetics exceeded three (maximum score was four). So, it appears that transport-related walking might only increase when the environment is perceived as very aesthetically pleasing. Moreover, the associations cannot be generalized across countries.

The associations between the walkability index and transport-related walking were curvilinear rather than linear and differed across study sites and genders. Associations were stronger in women and in Ghent and Seattle compared to men and in the Adelaide and Baltimore sites. In men and in Ghent, the associations weakened at higher levels of the walkability index, while in women and in the Seattle region in particular, a steeper increase in transport-related walking was found at higher levels of the index. In Baltimore and Adelaide, the associations were weaker, with a tendency for a steeper association at higher levels of the index. Perhaps higher levels of environmental support are needed to "encourage" women to walk for transport. The weakening of associations at higher levels of walkability in Ghent could be due to very high levels of mixed use requiring little walking, as appeared to be the case in a previous study of the Ghent region [25]. One conclusion emerging from present analyses is that the associations between physical environment attributes and transport-related walking are complex, suggesting that improving the activity-friendliness of an environment might have stronger effects on walking under certain environmental conditions and for women.

The curvilinear shape of some walkability-transport walking associations suggests that for some environmental perceptions, a ‘threshold’ needs to be crossed before transport-related walking will increase. Nonetheless, this threshold appears to be site- and gender-specific, so based on the present findings, no specific guidelines can be developed for optimal activity-enhancing environmental attributes that can be expected to generalize across countries. However, some attributes (e.g. residential density) were related to walking for transport in all three countries, suggesting there are generalizable principles at work. The shape of the environmental associations differed across behaviors. A linear association was found for transport-related cycling, so it appears that environmental changes across the entire range have the potential to increase the level of cycling, while a threshold may need to be exceeded in order to increase transport-related walking in adults. However, no definite conclusions can be drawn at this point. In order to further clarify the shape of these associations and reach more specific international guidelines for developing walkable and bikeable communities, further research should include more countries covering an even broader range of environmental variability.

The main strength of the present study was the assessment of large adult samples in three culturally- and environmentally-diverse countries. Consequently, larger variability in built environment characteristics was created than single-country study sites could provide. Within-country environmental variability was maximized by recruiting participants from high- and low-walkable neighborhoods of each site. Secondly, active transportation and perceived built environment attributes were measured using valid and reliable instruments. Limitations also need to be acknowledged. First, small European adaptations were applied to the Belgian version of the NEWS questionnaire, so only a limited number of comparable built environment items could be included in the analyses. Second, since European cities usually are denser than those in USA or Australia [44], systematic biases in reporting could have occurred. The between-country variance in environmental perceptions was rather limited, although considerable differences in objective environmental characteristics exist. These similar response patterns in the answers to the NEWS indicate that environmental perceptions may be relative and influenced by overall built environment/geographical characteristics within a country. Third, a cross-sectional design was used, precluding the determination of causality. Fourth, the interviewer-administered IPAQ was used in Belgium, while in the USA and Australia the self-administered version was used. Because adults tend to over-report their PA when completing the self-administered IPAQ [45], the present results may be biased. Fifth, the low response rates in the USA and Australia potentially could have introduced selection bias, though response rates were similar across neighborhood types in all countries. In Australia, participants were required to complete two lengthy surveys, six months apart, and direct financial incentives were specifically prohibited by the ethics review committee. In the USA, incentive payments were provided, but participants also needed to complete two waves of data collection. Although no incentives were provided in the Belgian study, the response rate was higher, possibly because participants only needed to complete one data collection wave and were visited at home instead of receiving a mailed survey [46]. Sixth, all measures used in analyses were self-reported. Seventh, there are environmental and cultural data other than what we have reported, which are relevant to understanding the similarities and differences between our study sites and the associations with active transportation. For example, it would be informative to take into account the nature and extent of road infrastructure and transport-mode share, gasoline prices or culturally-related attitudes towards physical activity.

## Conclusions

In summary, different neighborhood environmental correlates were found for walking compared to cycling. The traditional ‘walkability’ characteristics were of higher importance for transport-related walking, while perceptions related to the availability and quality of cycling and pedestrian facilities and parking difficulties were associated with cycling. Surprisingly, perceived aesthetics was a correlate of both of these transport-related behaviors, while other factors that have been associated with active transportation in previous research, like street connectivity and traffic safety [9,13,25,43], did not contribute to explaining active transportation in the present study. This might be partly due to the fact that composite environmental-perception indices were computed for the present study. The particular impact of certain characteristics was possibly overruled by other factors included in the indices. Most previous studies examined single environmental attributes and their associations with PA behaviors. However, the cumulative effects of multiple attributes are likely needed to have an impact on active transportation [18], so further research should keep focusing on identifying patterns of built environment correlates. Since this was one of the first studies to study cumulative effects of multiple environmental characteristics, no firm policy-related conclusions can be drawn yet. However, the results suggest it is likely to be necessary to focus on a combination of improving land use mix, walking/cycling facilities, aesthetics and reducing parking availability to increase transport-related cycling. For transport-related walking, our findings imply that it would be helpful to consider residential density, land use mix and aesthetics in relation to potential planning and public health initiatives.

Since data from only three Western countries were used in the present study, our results are somewhat exploratory. In order to formulate more definite conclusions, future studies including more countries covering a broader range of environmental and cultural variation are needed. An example of such a study is the International Physical Activity and the Environment (IPEN; http://www.ipenproject.org; Kerr et al., under review) study, which builds on the three studies included in this paper. The IPEN study will collect similar data in adults living in 12 countries worldwide, aiming to formulate international and country-specific recommendations on the contribution of built environment characteristics to explain physical activity in adults.

## Competing interests

The authors declare that they have no competing interests.

## Author’s contributions

All authors contributed to the design of different parts of the study. DVD, IDB and GC coordinated the Belgian data collection. JFS, TLC, BES, JK and LDF coordinated the USA data collection and NO the Australian data collection. EC did the data analyses and drafted the results section of the manuscript. DVD drafted the introduction, methods and discussion sections of the manuscript. All authors helped to draft the manuscript and revised the manuscript for important intellectual content. All authors read and approved the final manuscript.
